# Evaluation of the genotoxicity, cytotoxicity, and bioactivity of calcium silicate-based cements

**DOI:** 10.1186/s12903-024-03891-w

**Published:** 2024-01-20

**Authors:** Merve Esen, Yeliz Guven, Mehmet Fatih Seyhan, Handan Ersev, Elif Bahar Tuna-Ince

**Affiliations:** 1 Department of Pedodontics, Faculty of Dentistry, Istanbul University, Istanbul, Turkey; 2 Department of Molecular Biology and Genetics, Faculty of Art and Sciences, Istanbul Yeni Yuzyil University, Istanbul, Turkey; 3 Department of Endodontics, Faculty of Dentistry, Istanbul University, Istanbul, Turkey

**Keywords:** Genotoxicity, Cytotoxicity, Bioactivity, Cell viability, Angiogenic factors, ALP activity

## Abstract

**Background:**

As calcium silicate-based cements (CSCs) have found success in various vital pulp therapy applications, several new CSC products have emerged. This study aimed to assess the genotoxicity, cytotoxicity, and bioactivity of four CSCs by comparing the newly introduced materials Bio MTA+ and MTA Cem with previously studied materials, Biodentine and NeoMTA.

**Methods:**

Genotoxicity was evaluated using the micronucleus (MN) assay in human peripheral blood lymphocyte cells, measuring MN frequency and nuclear division index (NDI). Cytotoxicity was assessed in human dental pulp stem cells through the Water-Soluble Tetrazolium Salt-1 (WST-1) colorimetric assay. Bioactivity was determined by ELISA, measuring the levels of angiogenic and odontogenic markers (BMP-2, FGF-2, VEGF, and ALP). Statistical analyses included ANOVA, Dunnet and Sidak tests, and Wald chi-square test. (*p* < .05).

**Results:**

The MN frequency in the groups was significantly lower than that in the positive control group (tetraconazole) (*p < .05*). NDI values decreased with increasing concentration (*p < .05*). Bio MTA+ and NeoMTA showed decreased cell viability at all concentrations in 7-day cultures (*p < .01*). All materials increased BMP-2, FGF-2, and VEGF levels, with Biodentine and NeoMTA showing the highest levels of BMP-2 and FGF-2 on day 7. Biodentine displayed the highest VEGF levels on day 7. Biodentine and NeoMTA groups exhibited significantly higher ALP activity than the Bio MTA+ and MTA Cem groups by day 7.

**Conclusion:**

Bio MTA+ and MTA Cem demonstrated no genotoxic or cytotoxic effects. Moreover, this study revealed bioactive potentials of Bio MTA+ and MTA Cem by enhancing the expression of angiogenic and osteogenic growth factors.

## Introduction

Mineral Trioxide Aggregate (MTA), introduced to the market in the 1990s, has the ability to set in the presence of moisture, unlike most other dental products that exhibit optimal performance in dry environments [1]. In addition to its hydrophilicity, MTA exhibits several desirable features such as sealing ability, biocompatibility, bioactivity, and low solubility, making it highly applicable in various clinical situations in dentistry, including root-end fillings, perforation repairs, root canal treatments, vital pulp therapies, and regenerative endodontic procedures. MTA has undergone several modifications in its formulation to address drawbacks such as prolonged setting time, discoloration, and poor handling characteristics, while also enhancing the material’s antimicrobial properties, regenerative ability, and mechanical strength [1–3]. A more comprehensive term, ‘calcium silicate-based cements (CSCs), has been employed to describe these MTA-like materials, given their primary constituents of calcium and silicate [4].

Biodentine^™^ (Septodont, Saint-Maur-des-Fosses, France), a calcium silicate-based material, was introduced to the dental market in 2009 as a “dentine substitute” and has gained significant attention from researchers and clinicians due to its major advantages such as easier handling, shorter setting time, and reduced discoloration compared to MTA [5]. Studies have also proven that its mechanical strength and adhesion properties are superior to those of MTA [6, 7]. Moreover, it exhibits favorable biocompatibility and bioactivity [8]. Another tricalcium silicate-based material is NeoMTA^®^ (Nusmile Inc., Houston, TX; USA), which incorporates tantalum oxide as a radiopacifier instead of bismuth oxide, known for its association with the discoloration often attributed to traditional MTA [9]. Moreover, NeoMTA^®^ offers advantages such as faster setting and easier manipulation compared to traditional MTA [10].

Recently, two new CSCs, Bio MTA+ (CERKAMED, Poland) and MTA Cem (NexoBio, Chungcheongbuk-do, South Korea), were introduced to the market, offering the similar composition as the original MTA. Bio MTA+ incorporates hydroxyapatite and nanoceramic particles in its composition [11].

Biocompatibility refers to the material’s capacity to interact with vital tissues without inducing adverse tissue reactions, such as systemic or local toxicity, genotoxicity, mutagenicity, or carcinogenicity. CSCs come into contact with dental pulp cells (DPCs) and periodontal ligament cells (PDLCs) when employed for vital pulp treatments, apexification, perforation repair, and root-end filling [12, 13]. In vitro cytotoxicity assays, which constitute the initial step in evaluating the biocompatibility of materials, can be employed to test new endodontic materials [14].

Bioactivity, in its broadest sense, refers to a material’s capacity to actively engage with biological systems, promoting specific responses that contribute to tissue regeneration, repair, and overall therapeutic benefits in diverse applications. This concept goes beyond a limited focus on bone-bonding ability and encompasses a wide range of applications across various tissues and cellular environments. Bioactive materials have been characterized by their indirect effect to release ions or molecules, resulting in desired biological reactions, such as biomineralization, regeneration, antimicrobial effects, or an immune response [15–17]. Studies have shown that growth factors become entrapped in the dentin matrix during tooth development. These growth factors can be released either due to caries or as a result of specific materials applied in vital pulp treatments. Upon release, these growth factors from dentin play a significant role in recruiting and differentiating mesenchymal stem cells into an odontoblast-like phenotype, thereby enhancing their potential for mineralization. This reperative potential in vital pulp therapies can be ascribed to the indirect effect of material placement on dentin, such as CSCs [16, 18]. The biomineralization process is closely linked to local angiogenesis and vascularization events, and the interaction between osteoblastic and endothelial cells plays a crucial role in achieving positive outcomes in vital pulp therapy. Accordingly, it is required to investigate the impact of dental cements on the expression and release of angiogenic and osteogenic factors by precursor cells [19]. The assessment of bioactive properties can be performed by the quantitative analysis of odontogenic, osteogenic, and/or angiogenic markers or genes, followed by the evaluation of ALP activity and alizarin red staining [20].

Currently, there is limited information available on the biological properties of both Bio MTA+ and MTA Cem, particularly regarding biocompatibility and bioactivity. As a result, the primary objective of this study was to evaluate the genotoxicity, cytotoxicity, and bioactivity of recently introduced CSCs, Bio MTA+ and MTA Cem and to compare these characteristics with those of Biodentine and NeoMTA. The null hypothesis of the study was that there would be no significant differences among the tested materials concerning genotoxicity, cytotoxicity, and bioactivity.

## Materials and methods

### Tested materials and sample preparation

Bio MTA+, MTA Cem, Biodentine and NeoMTA were tested in this study. The composition of the test materials and their manufacturers are provided in Table 1. Each material was mixed as per the instructions outlined by the manufacturer in a laminar flow cabin under aseptic conditions. Each mixture was then inserted into a custom-made presterilized teflon mold with a diameter of 8 mm and a thickness of 2.0 mm and left undisturbed for four hours. Fifteen sample discs for each group were obtained. Upon complete setting, the surfaces of the specimens were exposed to 30 min of ultraviolet light to ensure sterility [21]. All discs were immersed in 50 ml Falcon tubes filled with 25 ml of DMEM (Thermo Fisher Scientific 31,885,023, MA, USA) and stored in an incubator at 37 °C for 24 h. The extracts of the materials were filtered with 0.22-µm pore size filter (Sartorius AG, Germany). The stock extracts were kept at -20 °C until experiments.

**Table 1:** Manufacturers and compositions of CSCs used in the study

Material	Manufacturer	Batch No	Composition
Bio MTA+	Cerkamed, Poland	2811221	Powder: Calcium oxide, hydroxyapatite, oxides of: silicon, iron, aluminum, sodium, potassium, bismuth, magnesium, zirconium; calcium phosphate. Liquid: Purified water, calcium catalyst.
MTA Cem	NexoBio, Chungcheongbuk-do, South Korea	MC180601	Powder: Tricalcium silicate, dicalcium silicate, tricalcium aluminate, bismuth oxide Liquid: Distilled water
Biodentine	Septodont, Saint-Maur-des-Fosses, France	B24589	Powder: Tricalcium silicate, Zirconium oxide, Calcium carbonate, Calcium chloride, polymer Liquid: Aqueous solution of calcium chloride and polycarboxylate.
NeoMTA	Nusmile Inc., Houston, TX; USA	2022051801	Powder: Tricalcium silicate, dicalcium silicate, tantalite, and minor amounts of calcium sulfate and tricalcium aluminate Liquid: water and proprietary polymers

### Genotoxicity analysis

Human peripheral blood lymphocyte cells were used for genotoxicity testing. Peripheral venous blood was collected from a healthy donor aged 28 years, who was a nonsmoker, had no chronic disease, and was not taking any medication or radiation. The blood donor gave informed consent to participate in the study and the study protocol received approval from the Ethics Committee of the Istanbul University Faculty of Dentistry (2018/28). The genotoxicity of the tested materials was evaluated using the micronucleus assay, which was carried out according to the protocol described by Akyil et al. [22]. Peripheral blood (0.3 ml) was added to sterile tubes containing 2.5 ml chromosome medium [23], which included phytohemagglutinin (PHA) as a mitogen. The samples were then incubated at 37 °C for 72 h.

Stock solutions of each material were prepared at three different concentrations (1, 10, 50 µg/ml) as recommended by the Organization for Economic Cooperation and Development (OECD) guidelines [24]. Tetraconazole was used as the positive control. At the 24th hour, all doses of the experimental and control groups were added to the tubes and incubated again. At the 44th hour of incubation, 8 µg/ml cytochalasin-B was added to the tubes to prevent cytokinesis and facilitate the formation of binucleate cells. After the incubation period, the tubes were centrifuged at 1200 rpm for 15 min, and the supernatants were removed. Five milliliters of 0.4% hypotonic KCL solution was then added slowly in drops. The centrifugation process was repeated. After removal of the supernatants, the cells in the tubes were treated with the first fixative, which was a mixture of glacial acetic acid, methanol, and 0.9% NaCl at a ratio of 1:5:6. After 20 min, the tubes were recentrifuged. The cells were then treated with the cold second fixative of glacial acetic acid and methanol at a 1:5 ratio. This procedure was repeated until the fluids in the tubes became clear. Finally, 1 ml of the samples in tubes was resuspended and dropped onto glass slides that were kept cold and cleaned with alcohol. After 24 h, the slides were dried and dyed with 5% Giemsa (Sigma-Aldrich, USA) dissolved in Sorenson buffer for 14 min. The slides were examined under an inverted microscope (Nikon Eclipse, Japan). Scoring of binucleated cells and micronuclei (MN) was performed based on the criteria outlined by Fenech [25]. The MN frequency was calculated as the number of MNs per 2000 binucleated cells. The effect of each tested CSCs on the cell cycle was assessed through the nuclear division index (NDI). An evaluation was conducted on 2,000 viable cells to determine the percentage of cells with 1, 2, 3, and 4 nuclei. NDI was calculated with the formula: (MI + 2xMII + 3xMIII + 4xMIV) / N; where, M1 to M4 denote the counts of cells with 1 to 4 nuclei, and N is the total number of intact cells scored [25].

### Cytotoxicity analysis

#### Cell culture

Commercially available human dental pulp stem cells (hDPSCs, Lonza Group, Basel, Switzerland) were used in the experiments to assess cell viability, angiogenic factor release and alkaline phosphatase (ALP) activity. The hDPSC growth medium was prepared as per the instructions outlined by the manufacturer. The cells were seeded in a 75 cm^2^ culture flask containing culture medium and kept in an incubator at 37 °C under standard conditions with 5% CO_2_. Upon reaching 80% confluency on the 5th day, the cells were subcultured.

During the passaging process, the medium was removed from the flasks and the cells were sequentially washed with 4 ml of HEPES followed by 2 ml of trypsin EDTA (Thermo Fisher Scientific, USA). After 5 min of incubation, the cells were successfully detached from the flask’s base. The detached cells were washed with 4 ml of trypsin neutralization solution (TNS) and then transferred into new sterile Falcon tubes. The tubes were centrifuged at 1500 rpm for 5 min and cultivated in fresh culture flasks. Cell cultures at the third passage were used for all experiments.

### Cell proliferation analysis

The cytotoxicity of the different CSC eluates was assessed using Cell Proliferation Reagent WST-1 (Roche Diagnostics, Mannheim, Germany) after 1, 3 and 7 days of culture. The hDPSCs were seeded in 96-well plates at a density of 1 × 10^4^ cells/well and exposed to various concentrations (12.5%, 25%, 50%, 75%, and 100%) of each material extract. Untreated cells were used as a negative control group. At each selected timepoint, 10 µL of the WST-1 reagent was added to each well, and the plates were incubated at 37 °C under 5% CO_2_ for 4 h. Finally, absorbance was measured at a wavelength of 450 nm with the reference wavelength set at 620 nm using a Multiskan Sky Microplate Spectrophotometer (ThermoFisher Scientific, Waltham, MA, USA).

### Bioactivity assessment

#### Measurement of angiogenic and osteogenic growth factors

The material extracts at 50% concentration were selected for the bioactivity assessment, based on the previous WST-1 cell proliferation results. Cells were seeded in three different 12-well plates at a density of 1 × 10^5^ cells/well. Following an overnight incubation, the culture medium was removed and at this stage, the cells in each plate were exposed to the diluted material extracts. Subsequently, cells in each plate were incubated 1, 3, and 7 days, respectively. On days 1, 3, and 7, the supernatant from each cell culture plate was collected, subjected to centrifugation, and subsequently stored at -80 °C for subsequent analysis. The amounts of Fibroblast Growth Factor 2 (FGF-2), Bone Morphogenetic Protein 2 (BMP-2) and Vascular Endothelial Growth Factor (VEGF) were quantified using their respective ELISA kits in accordance with each manufacturer’s protocol (Elabscience, Houston, Texas, USA; Invitrogen, ThermoFisher Scientific, MA, USA). The levels of BMP-2, FGF-2, and VEGF were determined via a microplate reader (ThermoFisher Scientific, MA, USA) at 450 nm.

#### Evaluation of ALP activity

ALP activity at 1, 3, and 7 days was measured in the culture supernatant, which was collected after exposure to 50% concentration of each material extract, using an ELISA kit (Elabscience, Houston, Texas, USA) adhering to the manufacturer’s recommended procedures. The absorbance was recorded at a wavelength of 450 nm and a standard curve of ALP concentration was generated. The results were then statistically evaluated.

### Statistical analysis

All experiments were conducted three times independently, with triplicate assessments for each. The findings of cytotoxicity and genotoxicity assays were analyzed with GraphPad Prism 6 software using one-way ANOVA. Post hoc verification was performed using Dunnet and Sidak tests. Data obtained from ELISA tests were analyzed using IBM SPSS V23 software. The interactions of the main effects, including time, concentration, and group, were examined using the generalized linear model method, and the Wald chi-square test was employed to compare the means. The significance level was set at *p* < .05.

## Results

### Genotoxicity analysis

**Fig. 1:**
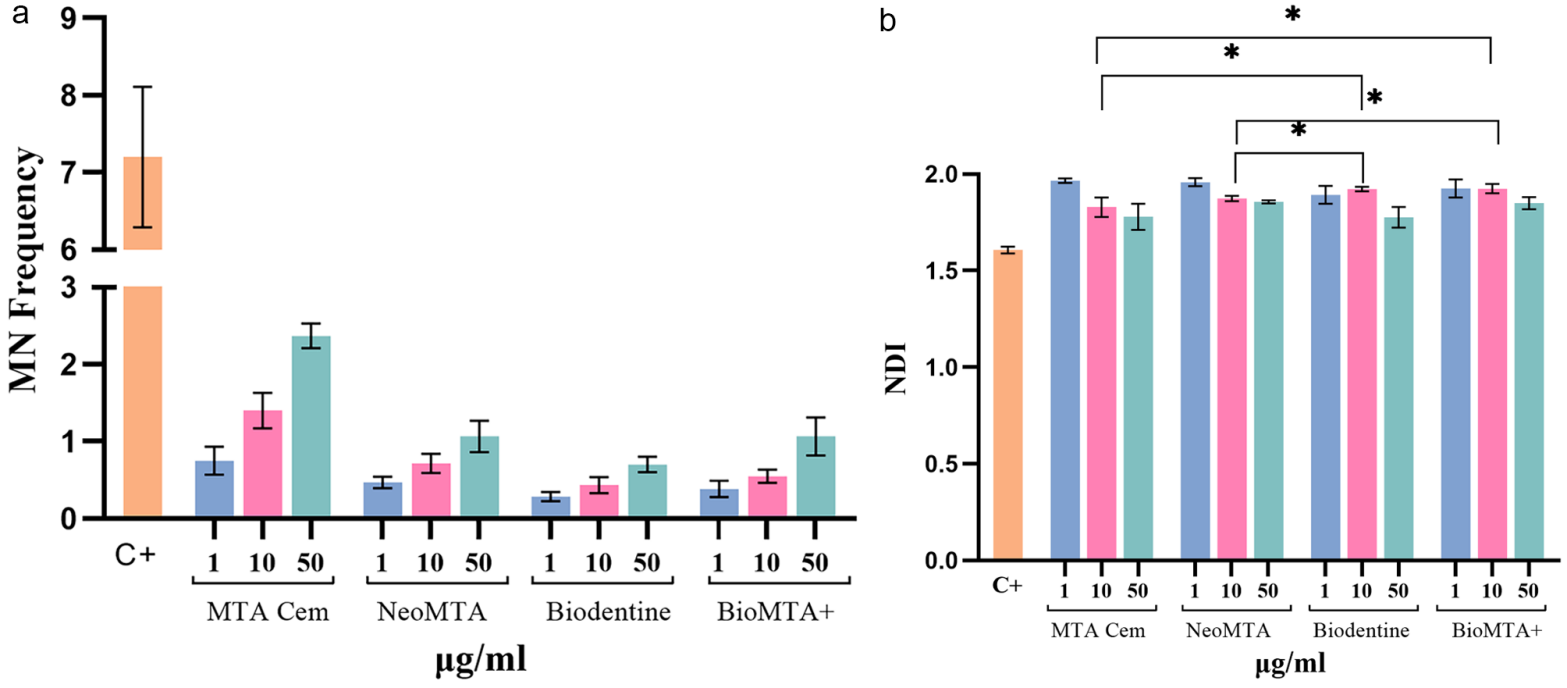
**(a, b).** MN frequency **(a)** and NDI **(b)** in human peripheral lymphocyte cells treated with three different concentrations of each material. (MN: Micronuclei, NDI: Nuclear Division Index C+: Positive control)

**Fig. 2:**
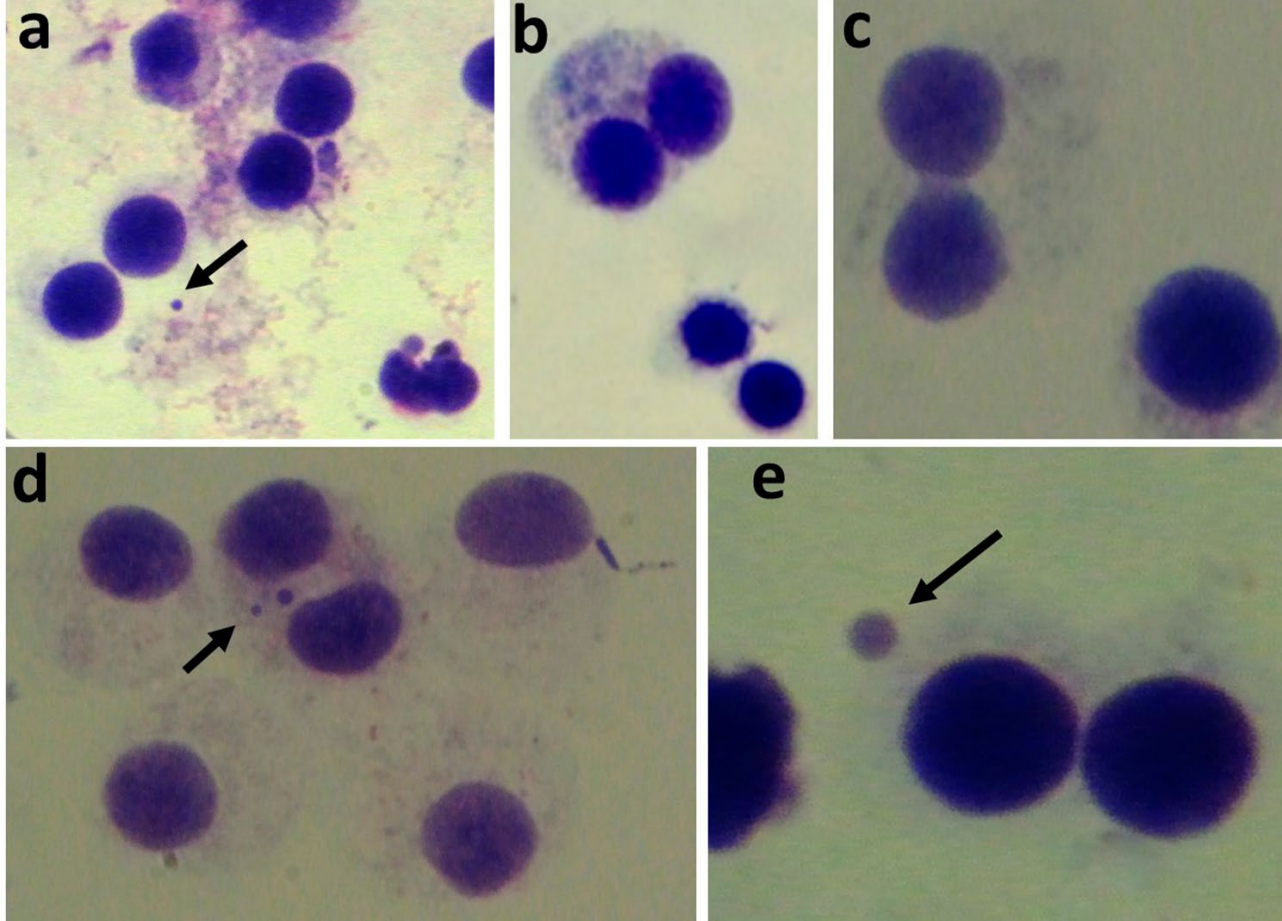
**a.** Control group showing binucleate cell with one micronucleus. **b.** MTA Cem 10 µg/ml group showing binucleate and mononucleate cells. **c**. NeoMTA 10 µg/ml group showing binucleate cell with a wide nucleoplasmic bridge and no micronuclei **(d)** Bio MTA+ 50 µg/ml group showing binucleate cells with two micronucleus **(e)** Biodentine 10 µg/ml showing binucleate cells with one micronucleus

The MN frequency and the NDI values for all concentrations (1 µg/ml, 10 µg/ml, 50 µg/ml) of each test group and the control group are presented in Fig. 1 (a,b). Representative images of binucleated cells stained with Giemsa in each group are depicted in Fig. 2. In all four groups, MN frequency increased with higher concentrations; however, these differences were not statistically significant (*p > .05*). Moreover, when compared to the positive control group, the MN frequency in each test group for all concentrations tested was found to be significantly lower (*p < .05*). No significant differences in MN frequency were observed among the test groups at the same concentrations (*p > .05*). At a concentration of 10 µg/ml, both the NeoMTA and MTA Cem groups exhibited significantly less nuclear division than the Bio MTA+ and Biodentine groups (*p < .05*). When NDI values of each test group were compared to that of the control group, statistical significance was found in all groups and all tested concentrations except Biodentine and MTA Cem at 50 µg/ml concentration.

### Cytotoxicity analysis

**Fig. 3:**
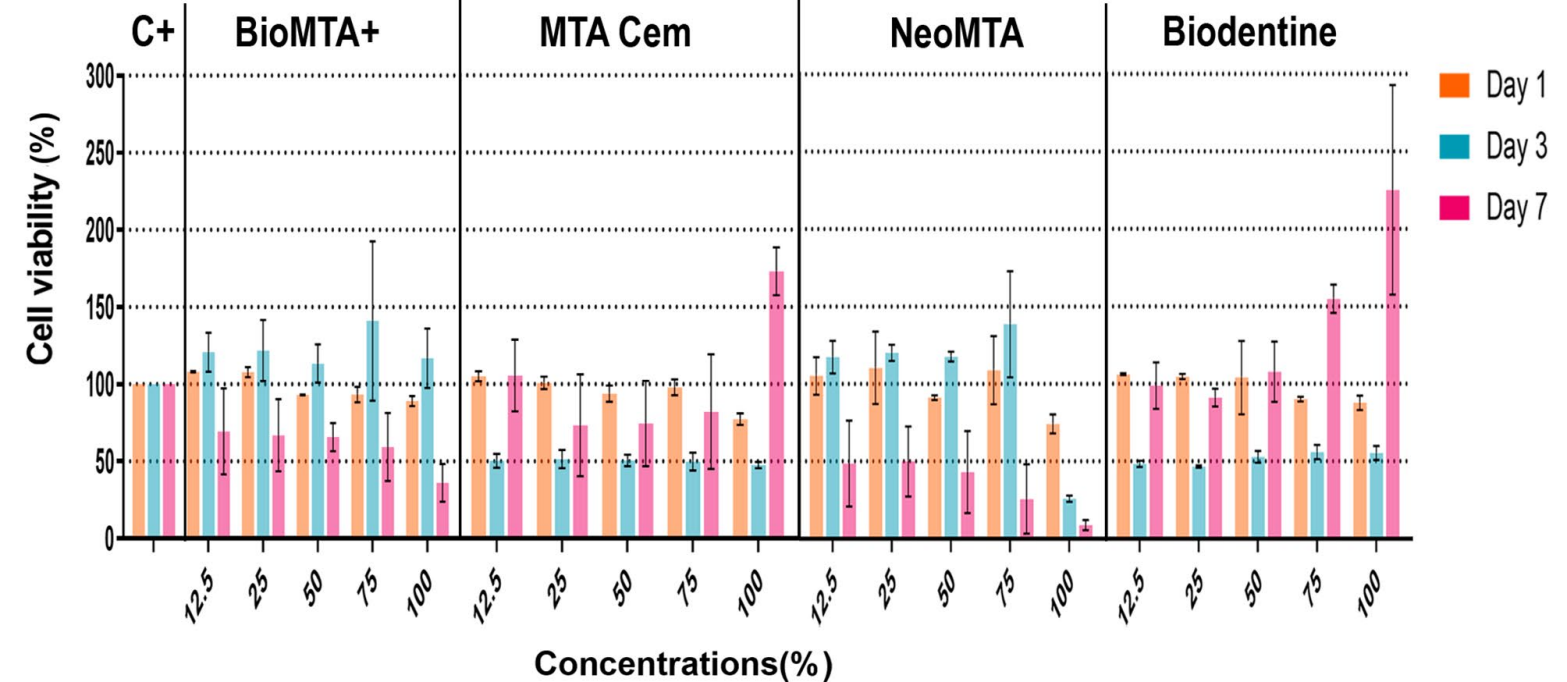
Cell viability of experimental and control groups at all concentrations on days 1, 3 and 7. Bars indicate mean standard deviation of the percentage compared to the control group. (C+: Positive control)

The cell viability of each group at all concentrations on days 1, 3 and 7 is shown in Fig. 3. After 1 day of culture, all four groups exhibited similar levels of cell viability compared to the control group. In 3-day cultures, both the Biodentine and MTA Cem groups demonstrated decreased cell viability compared to the control group at all concentrations (*p < .0001; p < .01*). The cell viability of Biodentine at 75% and 100% concentrations significantly increased in 7-day culture (*p < .0001)*. The Bio MTA+ and NeoMTA groups exhibited a significant decrease in cell viability at all concentrations in 7-day cultures (*p < .01*). However, they did not show any significant change in cell viability at all concentrations after 1 and 3 days of culture (*p > .05*).

### Angiogenic/osteogenic factor release

The BMP-2, FGF-2 and VEGF levels of each group on days 1, 3 and 7 are shown in Table 2. The ELISA results revealed a significant increase in BMP-2 levels in all groups over time (*p < .0001)*. On day 1, all experimental groups, including the control group, showed similar BMP-2 levels (*p > .05)*. However, by day 3, while there were no significant differences among the experimental groups, all of them exhibited significantly higher BMP-2 levels than the control group (*p < .0001)*. On day 7, MTA Cem had the lowest BMP-2 level among the experimental groups but remained significantly higher than that of the control group.

The levels of FGF-2 in all groups except the MTA Cem group were significantly increased over time (*p < .0001*). On day 1, there were no statistically significant differences in the levels of FGF-2 between any of the groups (*p > .05).* On day 3, the FGF-2 level of the Biodentine group was significantly higher than that of the NeoMTA and MTA Cem groups (*p < .0001*). On day 7, the Biodentine and NeoMTA groups demonstrated higher FGF-2 levels than the Bio MTA+ and MTA Cem groups (*p < .0001*).

All experimental groups, including the control group, showed similar VEGF levels on day 1 (*p > .05).* On day 3, the Biodentine group showed a significantly higher level of VEGF than the Bio MTA+ and NeoMTA groups (*p < .0001*). On day 7, the Biodentine group displayed the highest levels of VEGF among all groups (*p < .0001*).

**Table 2:** The BMP-2, FGF-2, VEGF, and ALP levels of each group on days 1, 3 and 7. BMP-2 levels significantly increased over time in all CSC groups, contrasting with the control group where they remained consistent. The highest FGF-2 levels were seen in Biodentine and NeoMTA groups on day 7. Bio MTA+ and NeoMTA exposure had no significant impact on VEGF levels at any time point, with the maximum VEGF level observed in the Biodentine group on day 7. All CSCs demonstrated elevated ALP activity compared to the control on days 3 and 7, with the highest ALP activity observed in the Biodentine and NeoMTA groups on day 7

Markers	Day	Groups					
		Bio MTA+	Biodentine	MTA CEM	NeoMTA	Control	Test statistics*
BMP-2	Day 1	180.1 ± 21.1^ab^	168.8 ± 31.9^ab^	132.4 ± 4.1^a^	147.4 ± 16.9^a^	90.3 ± 4.5^a^	6125.968
	Day 3	1142,5 ± 101.9^c^	1055.3 ± 46.1^c^	861.6 ± 112.1^c^	898.5 ± 58^c^	103 ± 7.4^a^	
	Day 7	2620.2 ± 320.1^d^	2438.3 ± 215.7^df^	1954.8 ± 118.8^h^	2295.6 ± 241.5^f^	156 ± 8.6^a^	
FGF-2	Day 1	35.8 ± 1.6^a^	38.5 ± 4.3^aef^	36.3 ± 3.9^ae^	33.8 ± 2.2^a^	31.3 ± 1.4^a^	2783.987
	Day 3	71 ± 13.7^bc^	77.7 ± 5.1^b^	54.8 ± 0.9^cfgi^	53.4 ± 0.9^efgi^	34 ± 1.9^a^	
	Day 7	119.5 ± 23.6^d^	147.4 ± 8.2^h^	68 ± 5.3^bci^	138.6 ± 16^h^	37 ± 1.5^ae^	
VEGF	Day 1	88.3 ± 16.6^abc^	117 ± 10.8^abcd^	96 ± 3.5^abc^	75.4 ± 1.7^bc^	61.9 ± 6.3^c^	4108.873
	Day 3	118.6 ± 6.6^abcd^	251.6 ± 27.3^f^	172.3 ± 9.5^adef^	115.9 ± 14.9^abcd^	79.1 ± 3.6^bc^	
	Day 7	152.4 ± 10.6^abde^	1050.9 ± 147.6^g^	363 ± 56.7^h^	88.8 ± 46.9^abc^	92.7 ± 2.2^abc^	
ALP	Day 1	175.8 ± 4.4^abcde^	261.3 ± 26.8^fhij^	121.2 ± 16.2^blnoprst^	144.3 ± 21.6^bcdlmnoprs^	82.1 ± 1.9^t^	1867.538
	Day 3	237.4 ± 16.8^fghijk^	293.6 ± 22^h^	247.1 ± 28.4^efhijk^	183 ± 17.2^acdegkm^	87.6 ± 1.8^ost^	
	Day 7	263.9 ± 23.4^fhj^	380.1 ± 22.9^q^	265.8 ± 10.7^hj^	382.9 ± 35.1^q^	91.7 ± 1.3^norst^	

*Wald Chi-Squared Test; Degree of freedom (df) = 29, a-i: For each marker, different letters represent significant differences between groups (*p < 001*)

### ALP activity analysis

The levels of ALP activity for each on days 1, 3 and 7 are shown in Table 2. On day 1, the ALP activity in all groups, except for MTA Cem, was significantly higher than that in the control group. Notably, the Biodentine group showed the highest ALP activity among all groups. On days 3 and 7, all groups exhibited significantly greater ALP activity than the control group. Additionally, by day 7, the Biodentine and NeoMTA groups demonstrated significantly higher ALP activity than the Bio MTA+ and MTA Cem groups.

## Discussion

In the present study, we aimed to compare the effects of four CSCs, namely Bio MTA+, MTA Cem, Biodentine, and NeoMTA, on genotoxicity, cell viability, the release of osteogenic/angiogenic factors, and the ALP activity in an in vitro setting. Our findings revealed variability in responses among the tested materials across most of the experimental conditions.

Genotoxicity tests were used investigate the impact of the tested material on the genetic integrity of cells. Numerous i*n vitro* and in vivo tests, including alkaline comet assay and the MN test, are used to identify genetic damage caused by materials, components, and drugs [26–28]. In the present study, the in vitro MN assay was chosen to determine the genotoxicity of the test groups, due to its ease of use, rapid scoring, and simplicity in culturing procedures [29]. The MN assay is based on the observation of whole chromosomes or their fragments being lost during cell mitosis, resulting in smaller nuclei or micronuclei that are not reintegrated into the nucleus after cell division. The OECD guidelines recommend using either cell lines such as CHL/IU, CHO, SHE, and V79 or human peripheral blood lymphocytes in the MN test [24]. In this study, we opted to use peripheral blood lymphocytes obtained from a single donor, as these cells can reflect the micronucleus frequency between tissues with cancer and lymphocytes.

Upon reviewing the available literature, a general agreement emerges indicating the favorable biocompatibility of calcium silicate-based materials. Studies have consistently yielded no evidence of genotoxic effects induced by CSCs in mammalian cells, with the exception of light-cured CSCs [27, 30, 31]. In the current study, the MN frequency in each experimental group was observed to be lower than that in the control group across all tested concentrations. The NDI in all groups was higher than that in the control group at all concentrations, except for the Biodentine and MTA Cem groups at a concentration of 50 µg/ml. These findings suggest that these cements do not exert any genotoxic effects on lymphocyte cells except at the highest concentrations of Biodentine and MTA Cem. Despite the contrasting results between MN frequency and NDI values concerning the highest doses of the Biodentine and MTA Cem groups, we reasoned that MN frequency provides a more accurate assessment of genotoxicity. This is based on the fact that MN frequency is a direct measurement of chromosome damage, unlike NDI, which primarily reflects damage in cell cycle resulting in a reduction in cell proliferation. Moreover, Fenech [25] noted that necrotic and apoptotic cells are not included in the total number of cells scored in NDI, leading to overestimated NDI values. Therefore, concerning genotoxicity, the null hypothesis was accepted.

The present study is the first to investigate the genotoxicity of MTA Cem. Therefore, it was not possible to compare the findings with those of other studies. Nonetheless, the biocompatibility was verified for MTA Cem, aligning with the outcomes generally reported for calcium silicate-based cements. To date, only one study has investigated the genotoxicity of Bio MTA+ through subcutaneous implantation in rats and and demonstrated good biocompatibility [32]. In the present study, the genotoxicity of Bio MTA+ was assessed in vitro through the MN assay, and no genotoxic effects on lymphocytes were demonstrated for all tested concentrations.

Human DPSCs exhibit multipotent characteristics, enabling them to proliferate, migrate to injury sites, and differentiate into odontoblast-like cells, promoting the formation of new dentin. When employed in vital pulp treatments, calcium silicate-based cements come into direct contact with dental pulp. As a result, evaluating the cytotoxicity and bioactivity of these materials on DPSCs is crucial for the repair of dentin-pulp complex tissues [33–35]. In the present study, the cytotoxicity of the materials was assessed using the WST-1 assay, which determines the number of viable hDPSCs based on their mitochondrial activity. Additionally, three dilutions of the conditioned medium for each material were used to mimic diverse clinical conditions. Notably, in vital pulp treatments, these materials may be applied to dentin layers ranging from 0.01 to 0.25 mm in thickness or directly in contact with exposed pulp. As such, the concentration of the material reaching viable pulp tissue might exhibit variations. Moreover, it is noteworthy that the presence of blood and lymphatic vessels in pulp tissue contributes to the dilution of substances [34, 36].

The analysis of WST-1 cell viability assay revealed variability in responses among the tested CSC materials and across various doses. Hence, the null hypothesis regarding the cytotoxicity of the materials was rejected in this study. In most previous studies, Biodentine and NeoMTA have been shown to be noncytotoxic to dental pulp cells, suggesting their potential as biocompatible materials in vital pulp therapy [34, 35, 37]. Based on our results, all tested cements exhibited comparable outcomes to the control group, indicating their cytocompatibility to DPSCs at all concentrations after 1 day of culture. However, in both the Biodentine and MTA Cem groups, cell viability steadily decreased after 3 days of culture. A similar pattern of results was obtained in a prior study by Youssef et al. [38], where Biodentine displayed significant cytotoxicity against DPSCs, reducing cell viability to 16% after 3 days of culture. It is noteworthy that the decline in cell viability on day 3 in our study was not as pronounced as in their study. Furthermore, in the Biodentine group, cell viability sharply increased after 7 days, reaching levels similar to or even higher than those in the control group. Additionally, in the present study, the undiluted extracts of NeoMTA exhibited significantly lower cell viability than the control group at 3 and 7 days of culture. This finding is in line with that of Kim et al. [39], as both our study and theirs reported 25% cell viability for the undiluted extracts of NeoMTA at 3 days of culture. The mechanisms underlying the observed cytotoxicity remain not fully elucidated. However, consistent with our findings, Tomas Catala et al. [35] reported lower cell viability for NeoMTA than for Biodentine. To address this discrepancy, they conducted an in-depth analysis of the chemical composition of the cement surfaces and their eluate extracts using SEM/EDS. Notably, they identified a 100-fold increase in strontium (Sr) levels in the eluates of NeoMTA compared to Biodentine. Additionally, silicon (Si) ions were detected on the surface of NeoMTA but were absent in Biodentine. They suggested that the lower cell viability of NeoMTA may be associated with the presence of ions such as Sr and Si in NeoMTA eluates, which are not found in Biodentine [35]. This explanation aligns with our own study results. Our findings demonstrate that Bio MTA+ exhibits biocompatibility, as it consistently maintains cell viability rates above 50% across all tested dilutions except for the undiluted extract after 7 days. This study is the first to investigate the cytotoxicity of MTA Cem and Bio MTA+. Biocompatibility was confirmed for both cements, aligning with the outcomes typically reported for calcium silicate-based cements in general.

The biomineralization process which is related to bioactivity of materials involves local angiogenesis and osteo/odontogenesis events. These events are regulated by several growth factors, among which BMP-2, FGF-2, and VEGF play significant roles [40]. BMP-2, a member of the transforming growth factor beta (TGF-β) family, plays a pivotal role in differentiating dental pulp stem cells into odontoblasts during vital pulp therapy, contributing to the formation of mineralized tissue bridges [41, 42]. The efficacy of MTA in inducing BMP-2 expression and calcification in hDPCs has been well established [43–47]. In the present study, BMP-2 expression in all test groups increased significantly over time. The Biodentine, Bio MTA+ and NeoMTA groups exhibited higher BMP-2 levels than the MTA Cem group on day 7.

VEGF, one of the most important angiogenic factors, plays a critical role in responding to pulp injury, leading to enhanced blood vessel permeability and angiogenesis during healing events [31]. A previous study showed that VEGF promoted the proliferation and differentiation of hDPCs into odontoblasts/osteoblasts, elevated the expression of genes commonly associated with osteogenesis/odontogenesis, and enhanced mineralization and the formation of tertiary dentin [48]. Previous studies have demonstrated that VEGF is upregulated by Biodentine in hDPSCs [38, 49]. To date, no studies on the levels of VEGF in Bio MTA+, NeoMTA, or MTA Cem have been reported. However, similar types of MTA brands such as Proroot MTA and MTA Angelus showed an enhancing effect on VEGF regulation [38, 50, 51]. In the present study, only Biodentine and MTA Cem upregulated VEGF on days 3 and 7 compared to the control. The VEGF level of Biodentine on day 7 was found to be significantly higher than those of the other groups. Given these findings, it can be inferred that Biodentine influenced VEGF expression to a greater extent, which is significant for angiogenesis and mineralization events.

An angiogenic growth factor, FGF-2, promotes wound healing and tissue repair [52]. FGF-2 has also been involved in odontogenesis [53] and the repair process of pulpal wounds [54, 55]. In vital pulp treatment, its expression contributes to pulpal healing process and tertiary dentin formation. In the current study, all groups showed increased FGF-2 expression on day 3 and day 7 when compared to the control. However, FGF-2 expression was significantly lower in the MTA Cem group than in the Bio MTA+, Biodentine, and NeoMTA groups. These data suggest that all materials contribute to angiogenesis and tertiary dentin formation by FGF-2 expression, but to a lesser extent for MTA Cem. In contrast to our findings, Olcay et al. reported no significant differences in FGF-2 and VEGF levels among their groups ProRoot MTA, Biodentine and Well-Root ST [40]. According to the previously discussed results of this study, the null hypothesis regarding the bioactivity was rejected due to the significant differences among the levels of BMP-, FGF-2 and VEGF in the tested materials.

ALP is a significant enzyme involved in the early stages of osteo/odontogenic differentiation. It plays a crucial role in tissue repair and healing after pulpal injury, as it promotes mineral deposition and tissue calcification [44]. In the present study, all groups had significantly higher ALP activity than the control groups on day 3 and day 7. Biodentine and NeoMTA induced a greater increase in ALP activity when compared with the Bio MTA+ and MTA Cem groups on day 7. In most studies, tricalcium silicate cements demonstrated the ability to enhance ALP activity and promote the osteogenic differentiation of stem cells [38, 44, 56]. The differentiation of progenitor cells into odontoblast- or osteoblast-like cells is critical for healing process in vital pulp therapy [57]. During their differentiation, osteo/odontoblasts secrete ALP and type I collagen, contributing to the maturation of the matrix and subsequent mineralization [58].

The present study had certain limitations. First, our evaluation of cell viability was primarily based on the WST-1 assay, and we did not examine cellular toxicity encompassing apoptosis and necrotic cell death pathways. These areas warrant further exploration. Another limitation may stem from the absence of prior reports that evaluated the bioactivity and cytocompatibility of Bio MTA+ or MTA Cem.

## Conclusion

Our study presented no genotoxic effects on human lymphocytes and no cytotoxicity on hDPSCs. Additionally, these materials exhibited bioactive properties by increasing the expression of angiogenic and osteogenic growth factors and enhancing ALP activity. Further studies are necessary to evaluate clinical outcomes and compare them with the in vitro findings.

## Data Availability

No datasets were generated or analysed during the current study.
